# Supplementary material for: Rapid reduction in global chromatin loop size after acute STAG2 reconstitution in human cancer cells
Source: bioRxiv. 2026 May 29:2026.05.26.727913. Preprint. [Version 1] doi: 10.64898/2026.05.26.727913 (PMC13232337; doi:10.64898/2026.05.26.727913)
Supplement: Supplementary file 1 [file NIHPP2026.05.26.727913v1-supplement-2.pdf]

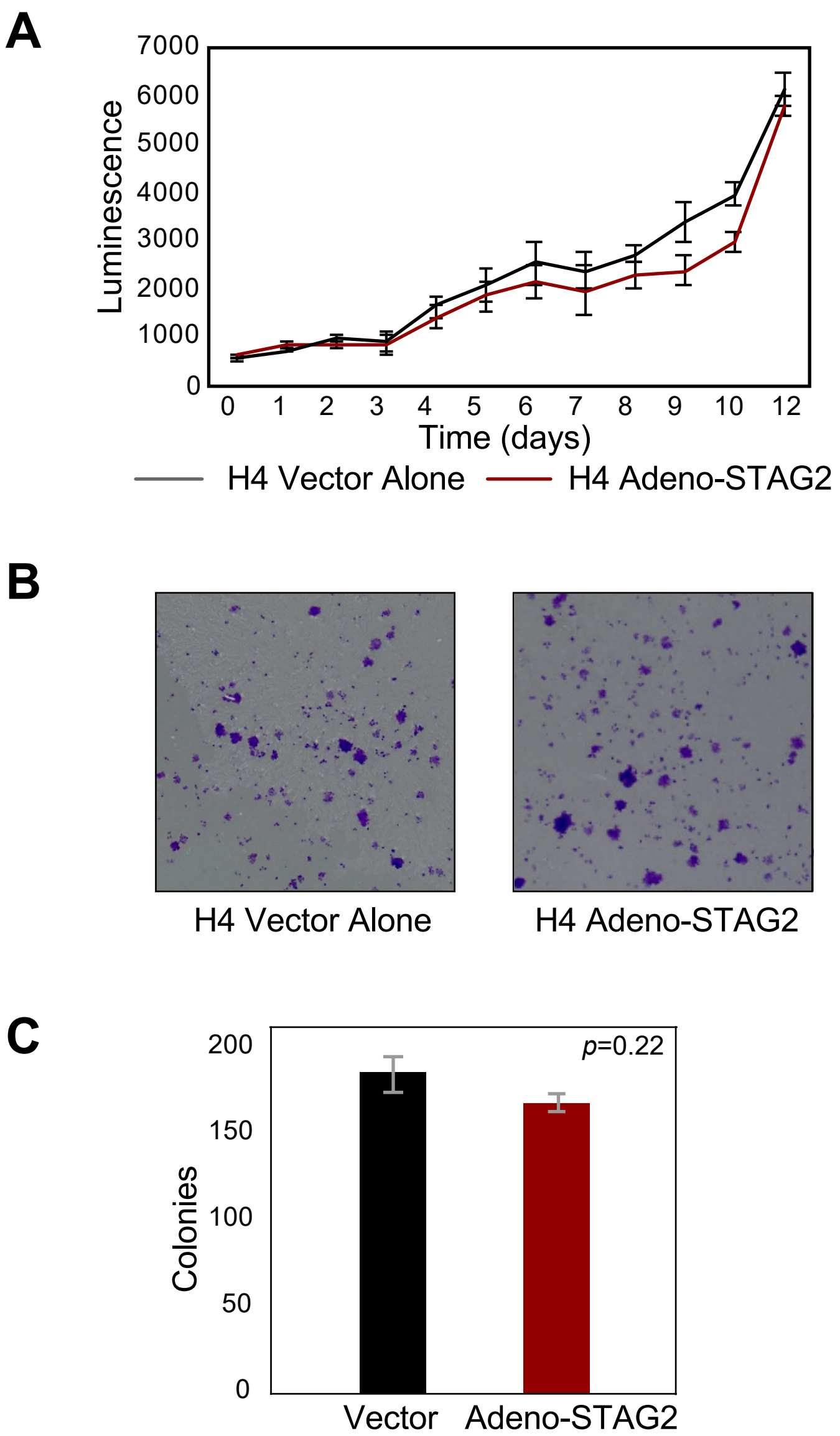


**Figure S1. Impact of adenoviral STAG2 reconstitution on proliferation and colony formation.** *A*, Proliferation of H4 cells infected with adeno-STAG2 or vector alone, measured using the CellTiter-Glo assay over 12 days. *B*,*C* Representative images and quantification of colony formation assays using the same cells as in (A). Error bars indicate S.D.

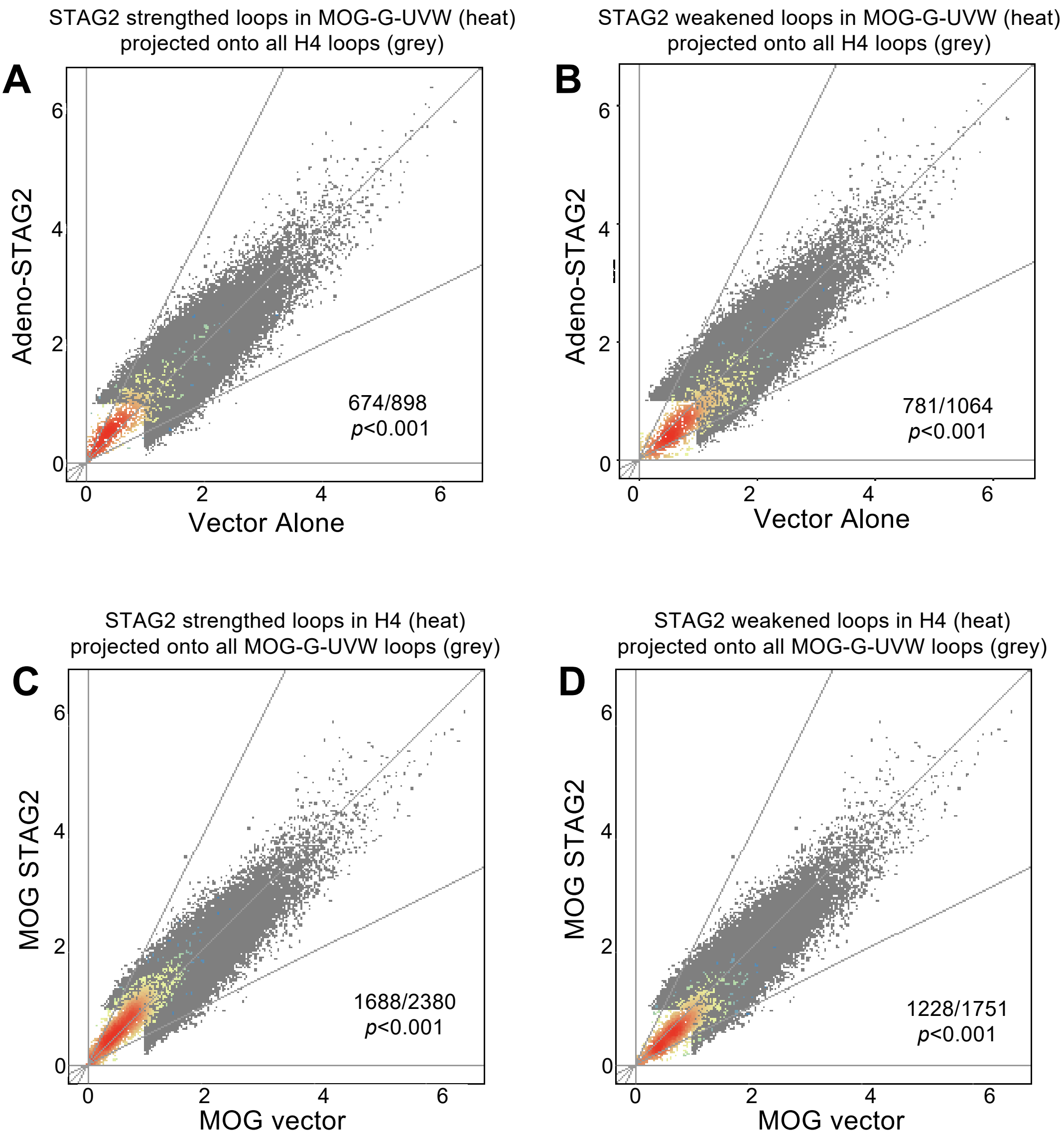


**Figure S2. Conservation of individual STAG2-regulated loops across cell lines.** A-D, Grey background scatter represents the intensity of the strongest ~300,000 chromatin loops in H4 cells (A,B) and MOG-G-UVW cells (C,D) infected with adenoviral vector alone (x-axis) and adeno-STAG2 (y-axis). Heat (red/yellow) represents the projection of STAG2 strengthed (A and C) and STAG2 weakened loops (B and D) from the converse cell line onto the grey scatter. The fraction of the projected loops showing consistent regulation in the two cell lines is indicated together with a *p*-value.

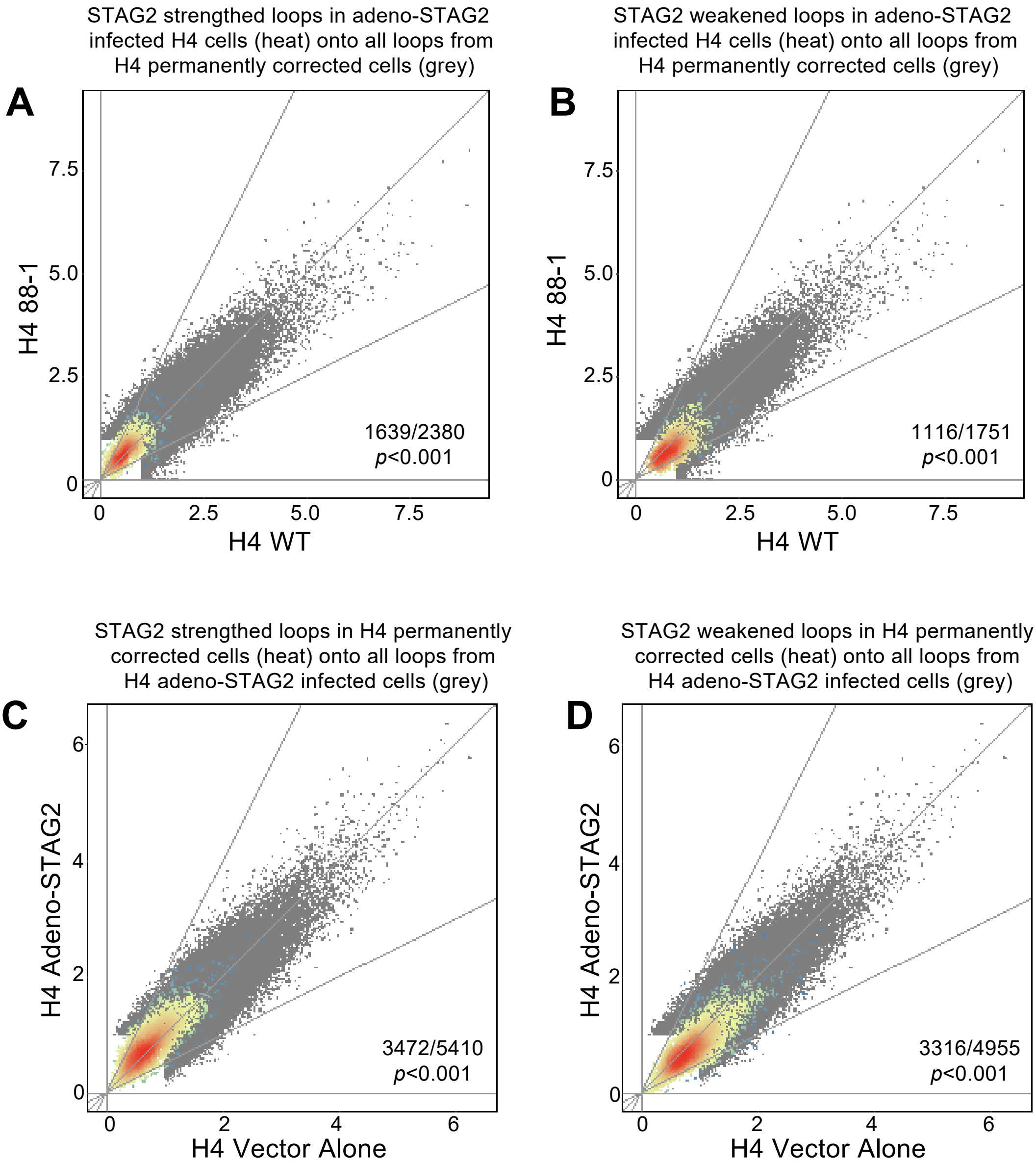


**Figure S3. Conservation of individual STAG2-regulated loops in transiently and permanently STAG2-corrected H4 cells.** A-D, Grey background scatter represents the intensity of the strongest ~300,000 chromatin loops in permanently STAG2-corrected H4 cells (A,B) and adeno-STAG2 infected H4 cells (C,D). Heat (red/yellow) represents the projection of STAG2 strengthed (A and C) and STAG2 weakened loops (B and D) from the converse cell system onto the grey scatter. The fraction of the projected loops showing consistent regulation in the experimental systems is indicated together with a *p*-value.

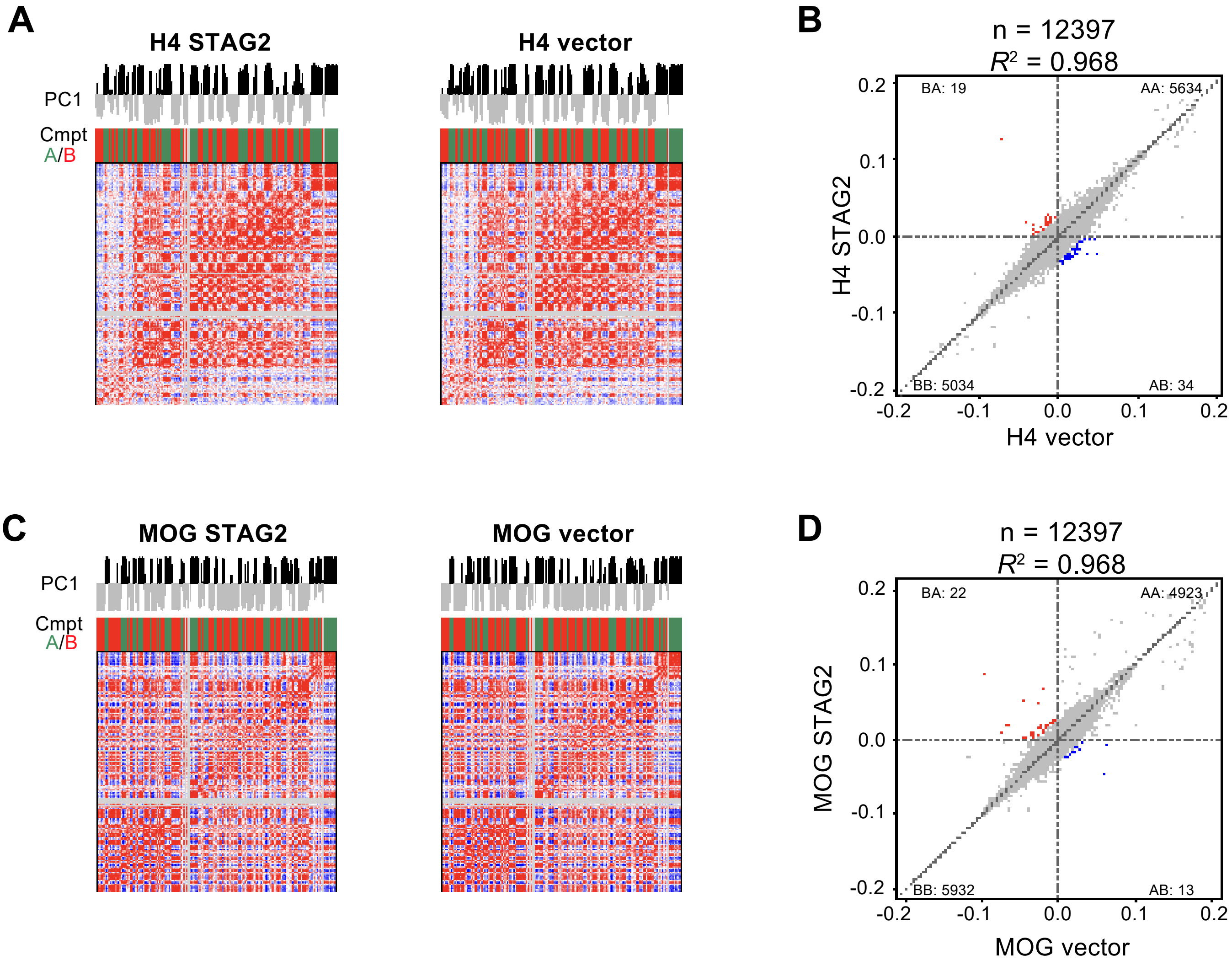


**Figure S4. Impact of rapid STAG2 reconstitution on A/B compartment assignments in H4 and MOG-G-UVW cells.** *A* and *C*, representative heatmaps of chromosome 2 showing Hi-C correlation matrices at 250-kb resolution for H4 cells (A) and MOG-G-UVW cells (C) infected with adeno-STAG2 or vector alone. Principal Component 1 (PC1) values define the A (green) or B (red) compartment status of individual 250-kb bins. *B* and *D*, scatterplot analysis depicting the effect of rapid STAG2 reconstitution on compartment assignments in H4 cells (B) and MOG-G-UVW cells (D). Bins undergoing compartment switches ($p<0.01$) are indicated in red (B to A) and blue (A to B). Coefficient of determination ($R^2$) and the total number of bins (n) are indicated.

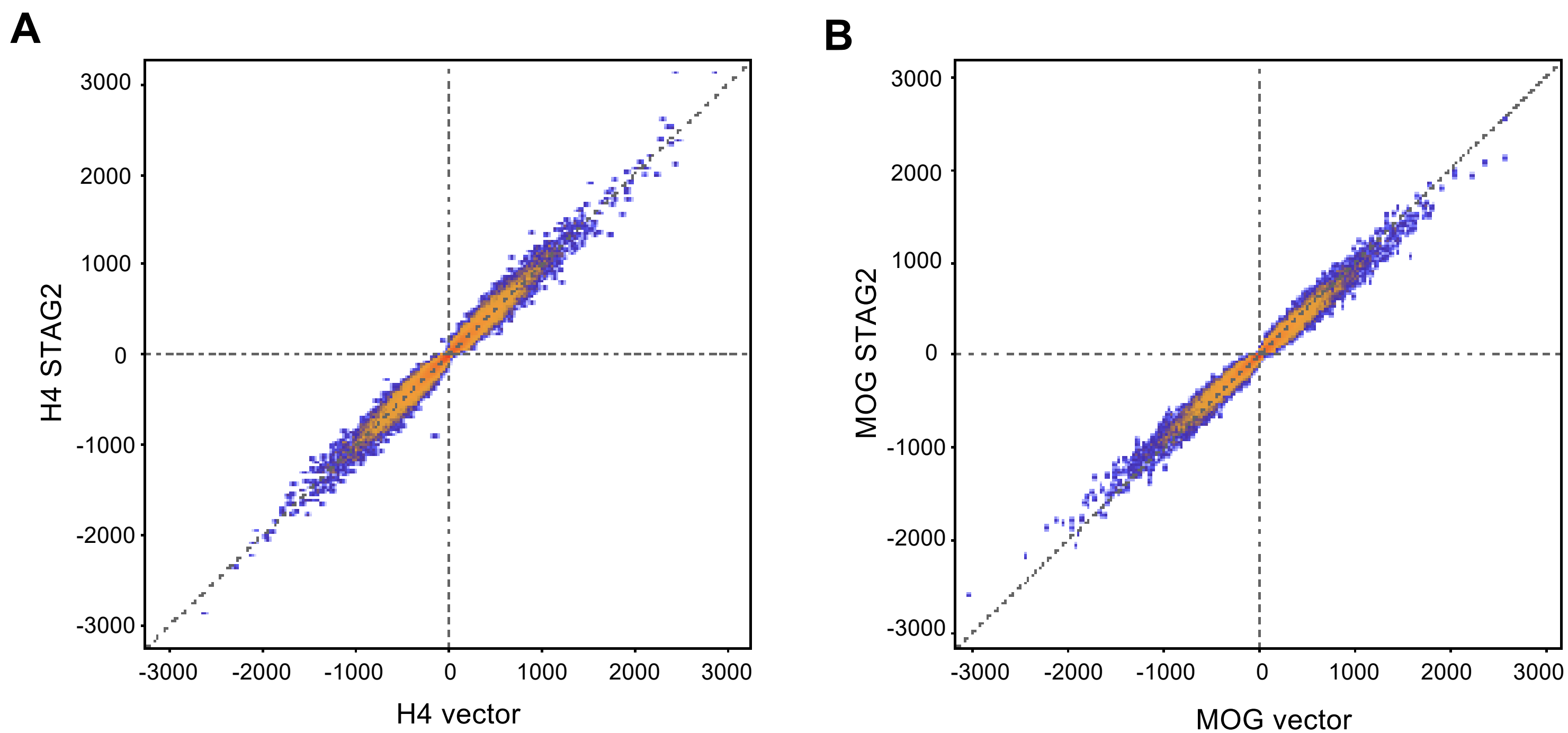


**Figure S5. Impact of rapid STAG2 reconstitution on topologically associating domains (TADs) in H4 and MOG-G-UVW cells.** Scatterplots depicting the effect of rapid STAG2 reconstitution on directionality indices (DI; a measure of TAD boundary strength) in H4 (A) and MOG-G-UVW (B) cells infected with adeno-STAG2 or vector alone.

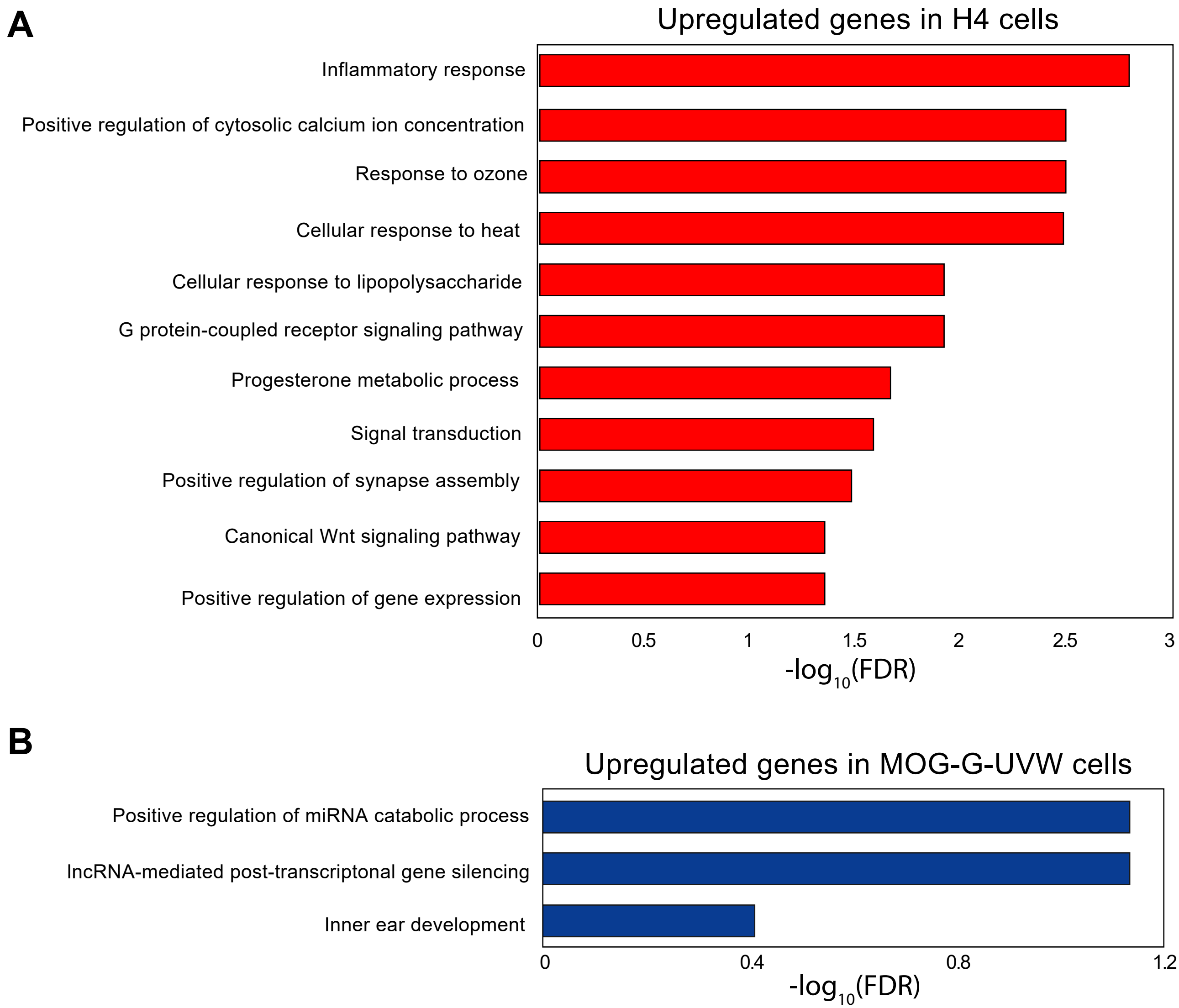


**Figure S6. Gene Ontology (GO) analysis of STAG2-regulated genes.** *A*, GO terms overrepresented in genes upregulated (FC>1.5) in H4 cells after infection with adeno-STAG2 (FDR<0.05). No GO terms overrepresented in downregulated genes met FDR<0.05 and therefore are not shown. *B*, Same as A for MOG-G-UVW cells.

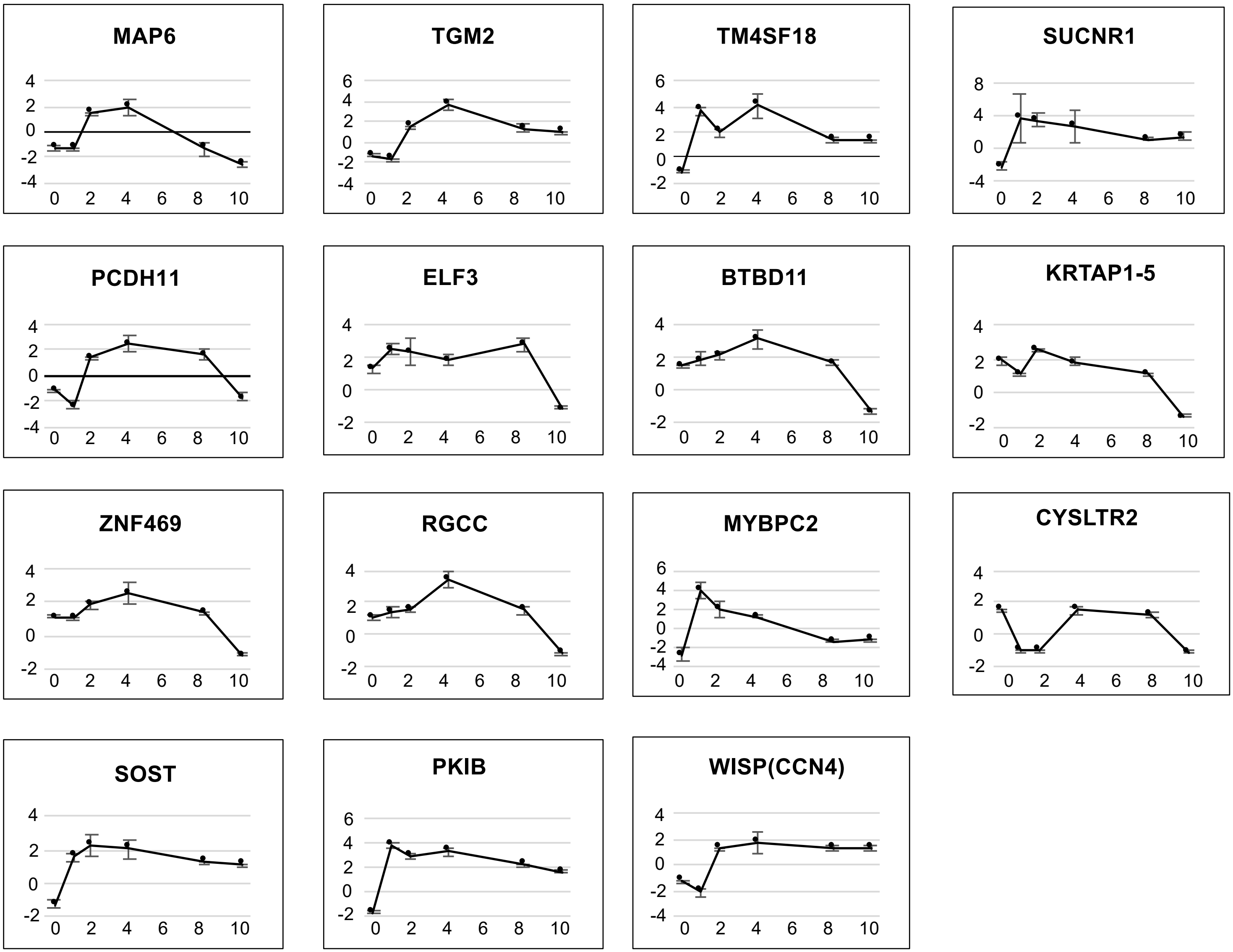


**Figure S7. Induction kinetics of STAG2-regulated genes.** Expression of STAG2 regulated genes at the indicated days following infection of H4 cells with adeno-STAG2 or vector alone, as measured by qRT-PCR. Fold change indicates the increase in expression after infection with adeno-STAG2 normalized to expression in cells infected with vector alone. Error bars represent S.D.

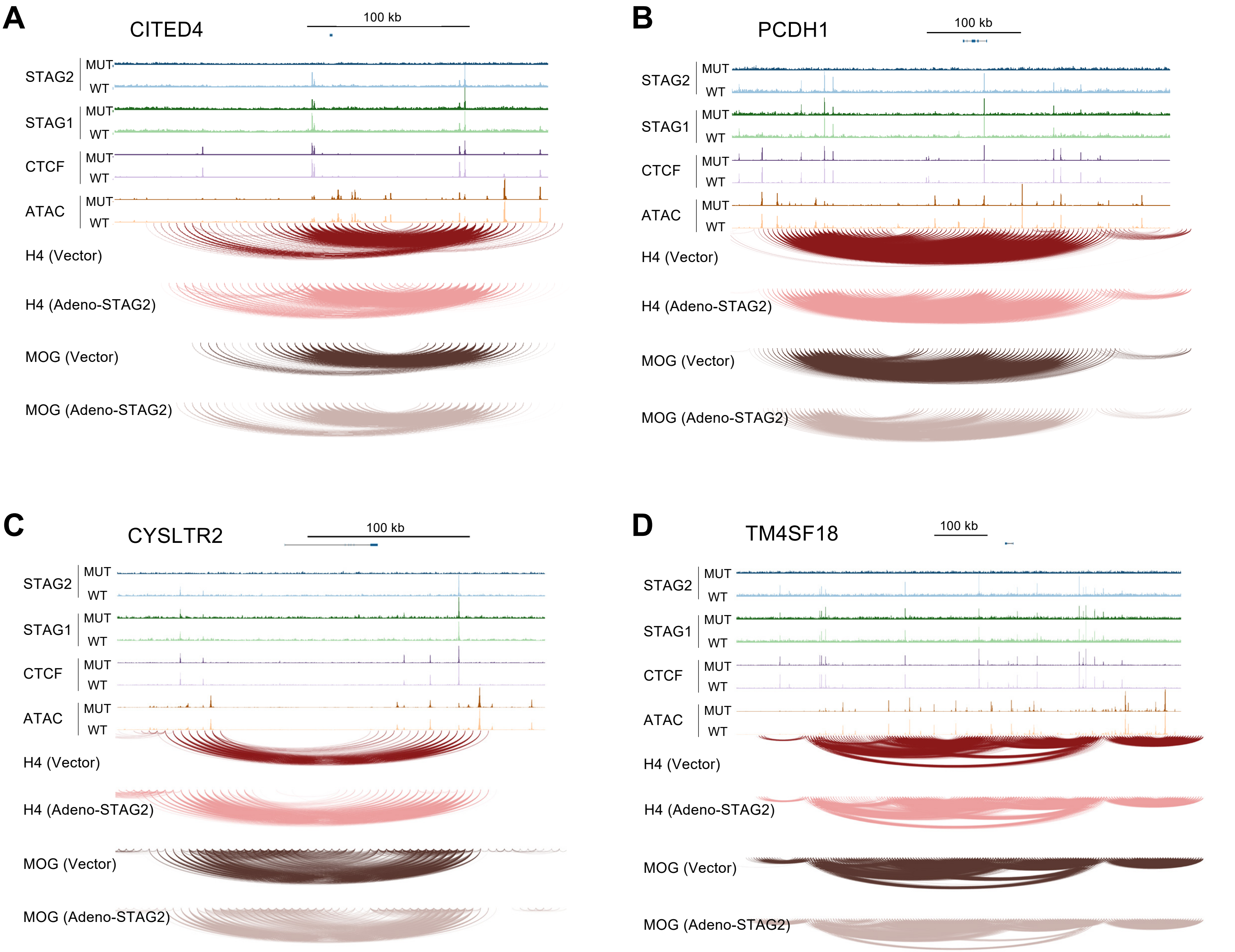


**Figure S8. Integrative genomics surrounding the CITED4, PCDH1, CYSLTR2, and TM4SF18 loci.** Schematic representation of the location and intensity of STAG2, STAG1, and CTCF chromatin binding sites by ChIP-seq (top); regions of open and closed chromatin by ATAC-seq (middle); and chromatin loops (bottom) surrounding the (*A*) CITED4 (chr1:41,180,006–41,474,755), (*B*) PCDH1 (chr5:140,963,663–141,476,413), (*C*) CYSLTR2 (chr13:49,111,678–49,401,750), and (*D*) TM4SF18 (chr3:148,489,014–149,408,708) loci.

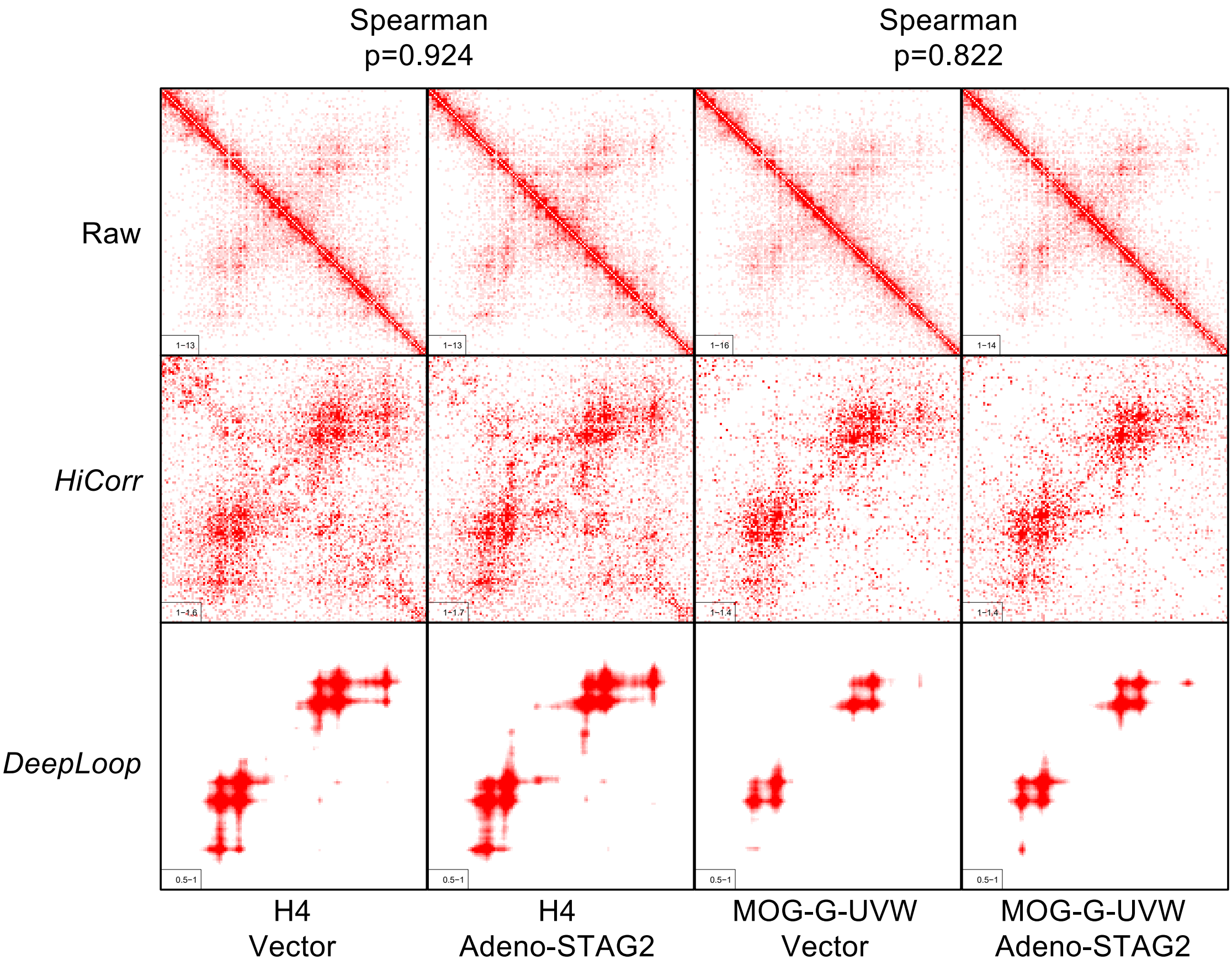


**Figure S9. EFEMP1 locus Hi-C contact maps.** Representative heatmaps showing normalized loop intensity at the EFEMP1 locus (chr2:55,797,886–56,456,442) in H4 and MOG-G-UVW cells. Data are presented for cells 36 hours post-infection with either adeno-STAG2 or an empty vector control. Maps are shown at 5-kb resolution following noise correction (HiCorr) and enhancement (DeepLoop). High Spearman correlation coefficients between adeno-STAG2 and vector-only conditions (0.924 for H4; 0.822 for MOG-G-UVW) indicate that chromatin loop structure and intensity surrounding EFEMP1 remain largely unchanged by STAG2 expression.

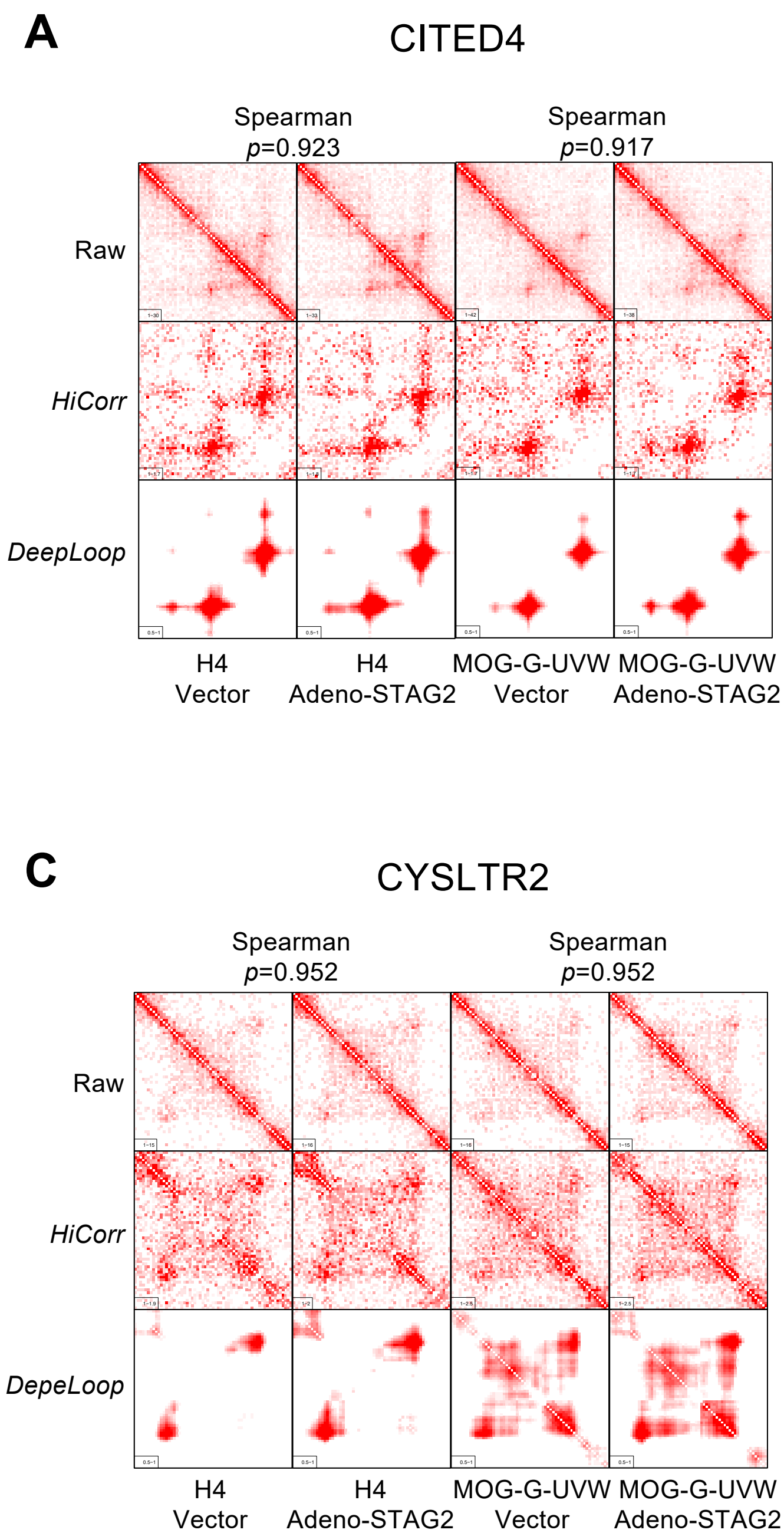


**Figure S10. Hi-C contact maps for the CITED4, PCDH1, CYSLTR2, and TM4SF18 loci.** Representative heatmaps showing normalized loop intensity at 5-kb resolution for four genomic regions: CITED4 (chr1:41,180,006–41,474,755), PCDH1 (chr5:140,963,663–141,476,413), CYSLTR2 (chr13:49,111,678–49,401,750), and TM4SF18 (chr3:148,489,014–149,408,708). Data are shown for H4 and MOG-G-UVW cells 36 hours post-infection with adeno-STAG2 or an empty vector control. Maps were processed via noise correction (HiCorr) and enhancement (DeepLoop). High Spearman correlation coefficients across all regions in both H4 and MOG-G-UVW lines (e.g., 0.924 for H4; 0.822 for MOG-G-UVW) indicate that chromatin architecture and loop intensity remain largely unchanged by STAG2 expression.

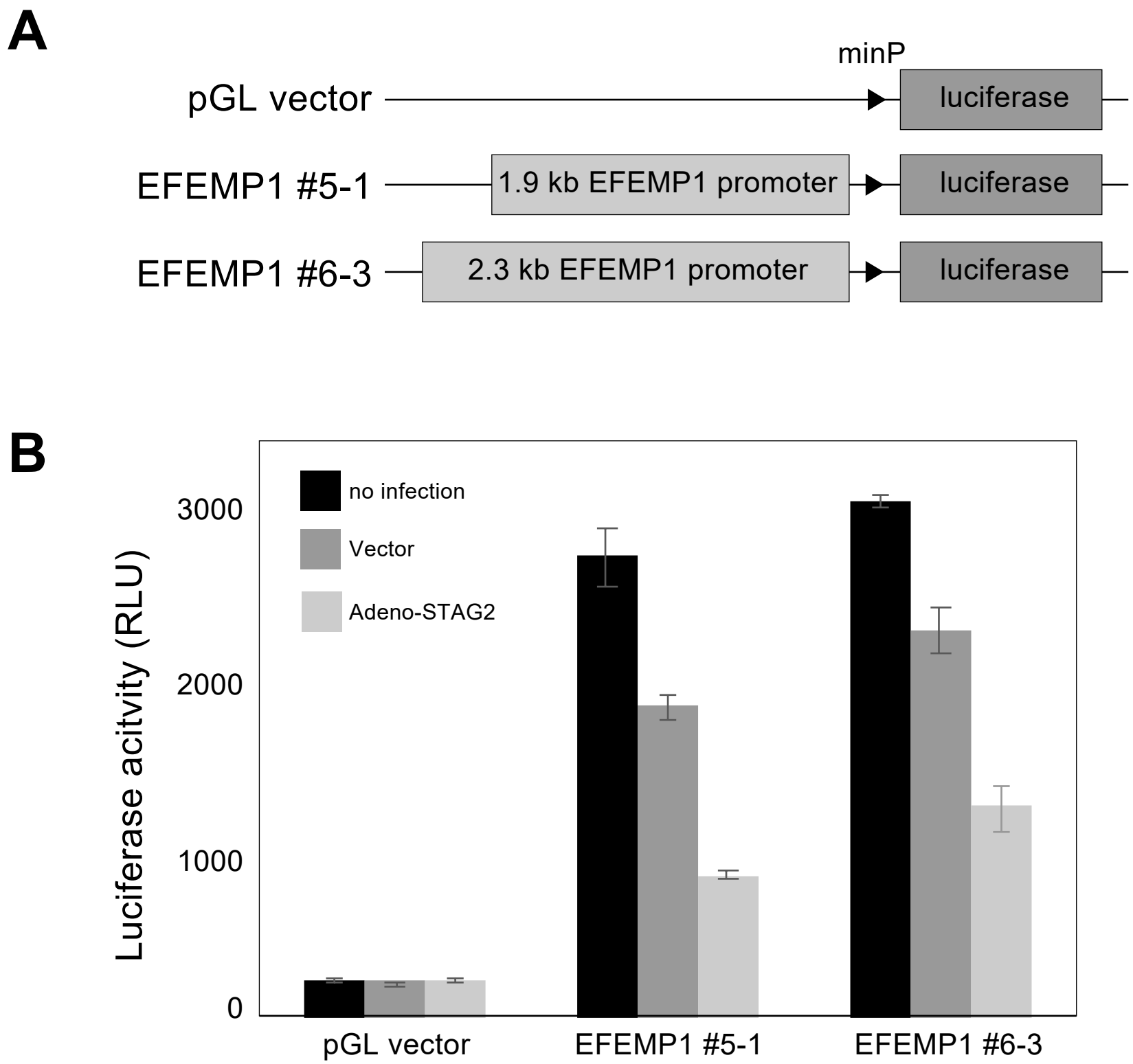


**Figure S11. EFEMP1 luciferase reporter assay.** *A*, Schematic representation of luciferase reporter constructs driven by the EFEMP1 promoter. *B*, MOG-G-UVW cells were transfected with the EFEMP1 promoter-luciferase reporter constructs and subsequently infected with either adeno-STAG2 or an empty vector control. Luciferase activity was measured 24 hours post-infection and normalized to Renilla luciferase activity. Data represent the mean ± S.D.
